# Cognitive behavioural therapy for insomnia decreases the discrepancy between objective and subjective measures of sleep

**DOI:** 10.47626/2237-6089-2024-0819

**Published:** 2025-11-05

**Authors:** Lauren E. Cudney, Sheryl M. Green, Randi E. McCabe, Benicio N. Frey

**Affiliations:** 1 Neuroscience & Behaviour, McMaster University Department of Psychology Hamilton ON Canada Department of Psychology, Neuroscience & Behaviour, McMaster University, Hamilton, ON, Canada.; 2 Research Institute of St. Joe's Hamilton Women's Health Concerns Clinic Hamilton ON Canada Women's Health Concerns Clinic, Research Institute of St. Joe's Hamilton, Hamilton, ON, Canada.; 3 McMaster University Department of Psychiatry & Behavioural Neurosciences Hamilton ON Canada Department of Psychiatry & Behavioural Neurosciences, McMaster University, Hamilton, ON, Canada.; 4 Research Institute of St. Joe's Hamilton Mental Health and Addictions Program Hamilton ON Canada Mental Health and Addictions Program, Research Institute of St. Joe's Hamilton, Hamilton, ON, Canada.

**Keywords:** Insomnia, cognitive behavioural therapy for insomnia, actigraphy, subjective-objective sleep discrepancy

## Abstract

**Objective:**

Individuals with insomnia disorder often exhibit differences between reported experiences of sleep and objectively measured sleep parameters; however, the implications of this subjective-objective sleep discrepancy during treatment remains unclear. The aim of this study was to investigate the impact of cognitive behavioural therapy for insomnia (CBT-I) on the discrepancy between objective and subjective measures of sleep, and to assess whether changes in clinical variables such as depression, anxiety, fatigue, and beliefs about sleep, were related to changes in discrepancy.

**Methods:**

Twenty-five participants with insomnia disorder were enrolled in group CBT-I. Sleep measures were continually sampled from baseline until 2 weeks post-treatment with both objective (i.e., actigraphy) and subjective (i.e., sleep diary) methods.

**Results:**

The subjective-objective discrepancy significantly decreased from baseline early on in treatment (following the second session) and were maintained at post-treatment for sleep onset latency, wake after sleep onset (WASO) and sleep efficiency (SE). Total sleep time (TST) discrepancy and misperception decreased from baseline to post-treatment. Improvement in depression symptoms, fatigue symptoms, and negative beliefs about sleep were significantly correlated with the decrease in the discrepancy for WASO and SE.

**Conclusion:**

These findings suggest that CBT-I resolves the mismatch between objective and subjective sleep parameters early in treatment for adults with insomnia. Sleep misperception improved from underestimating to accurately estimating TST. Improvement of psychological symptoms were related to decrease in sleep discrepancies across treatment. Future research is needed to explore how feedback on objective and subjective sleep discrepancy may impact sleep perception across treatment with CBT-I.

## Introduction

Insomnia disorder is characterized by persistent difficulties with falling asleep, maintaining sleep, and/or early morning awakenings accompanied by daytime impairments for at least 3 months^1^ and is diagnosed based on subjective reporting of sleep disturbance. Sleep questionnaires gather one's retrospective views on sleep, which are susceptible to the influence of one's mood and anxiety.^2^ A prospective sleep diary provides a comprehensive assessment of sleep experiences and self-reported insomnia symptoms. This includes time into and out of bed, amount of time taken to fall asleep (sleep onset latency [SOL]), sleep duration (total sleep time [TST]), length of awakenings after sleep onset (wake after sleep onset [WASO]), and subjective ratings of sleep quality, and is less biased than retrospective questionnaires about sleep.^3^ As a result, subjective sleep parameters may differ based on the method by which they are reported (e.g., sleep diary versus self-report questionnaire).^4^ Sleep duration, for example, was significantly longer when reported on a sleep diary compared to questionnaires in a longitudinal cohort of adults. To add to this, self-reported insomnia symptoms were associated with greater perceived differences between sleep diary and questionnaire data.^4^

Although subjective experiences of sleep are the basis of an insomnia disorder diagnosis, objective measures of sleep, including polysomnography (PSG) and actigraphy, have increasingly been employed to aid in the diagnosis of insomnia and to rule out other sleep disorders.^5^ PSG includes continuous measurement of electrical activity throughout the night, often done in a lab, which can be costly and in conditions that are quite different from at-home sleep. Wrist-worn actigraphy employs accelerometry to measure movement in order to estimate sleep parameters.^6^ Actigraphy is increasingly used in the sleep medicine field as a cost-effective and convenient tool for measuring sleep variables, which can collect objective sleep data in one's own home environment.

Interestingly, individuals with insomnia disorder commonly describe having sleep quantity and quality issues, even when objective sleep measures appear normal.^7^ This discrepancy between self-report or subjective experiences of sleep and objectively measured sleep parameters has been referred to in different ways over time including, "subjective insomnia," "paradoxical insomnia,"^8^ or "sleep-state misperception."^9^ Paradoxical insomnia was differentiated as a subtype of insomnia in previous versions of the International Classification of Sleep Disorders, but this has been controversial and it is no longer seen as a separate category of insomnia nor conceptualized as being significantly different than those whose sleep disturbance is captured objectively.^10^ This subjective-objective discrepancy may occur within a continuum of insomnia disorder, rather than as a separate subcategory of the disorder.^9^ The discrepancy between subjective and objective sleep measures may contribute to the trivialization and under-treatment of insomnia disorder.^9^ Rather, misperception of sleep has been proposed as a "prodrome" for the development of a more serious objective sleep deficit.^9^ The theoretical mechanisms of this transition from misperception of sleep to insomnia disorder that the discrepancy increases distress related to sleep and results in increased arousal. This suggests that sleep misperception itself may be an important target for treatment.^9^

Cognitive behavioural therapy for insomnia (CBT-I) is the first line treatment for chronic insomnia that targets the cognitive and behavioural factors that contribute to persistent sleep difficulties.^11^ Important components of CBT-I include psychoeducation, stimulus control, time-in-bed restriction, and cognitive restructuring.^12^ CBT-I has been shown to be effective at improving self-reported insomnia symptom severity, as well as sleep parameters measured with a sleep diary.^13^ A recent meta-analysis revealed that PSG sleep measures did not change across CBT-I treatment, whereas objective sleep parameters measured with actigraphy had mixed results including a small improvement in SOL and a reduction in TST with a moderate effect size.^14^ There are a number of studies that have investigated subjective and objective measures separately following CBT-I, but relatively few studies that look at the impact of treatment on the discrepancy between objective and subjective sleep parameters.^15^ One such study by Lovato et al.^16^ compared those with insomnia with objective short sleep vs. objective normal sleep duration at baseline. However, a recent study found that actigraphy and PSG identified the "short sleepers" differently, with only 51% consistency between the two objective sleep methods.^17^ Interestingly, all participants showed significant improvement in self-report insomnia symptoms and diary-reported sleep parameters following CBT-I regardless of whether they were identified as having short sleep duration or not, which suggests that this is not a reliable or useful distinction.^17^

The studies that have evaluated change in sleep perception across treatment have primarily focused on older adult populations only.^16,18-21^ To date, only one study has investigated subjective-objective sleep discrepancy across treatment with CBT-I in a population that included young and middle-aged adults as well.^22^ Janků et al.^22^ looked at session-by-session changes in subjective-objective sleep discrepancies in adults across group CBT-I. They identified that the discrepancies for SOL decreased following CBT-I, but that the discrepancy in WASO did not significantly change.^22^ This study identified that their total population was overall quite accurate with estimating TST at baseline and that there was a shift toward overestimating TST by post-treatment due to a decrease in objective TST and no changes in subjective TST.^22^ Since many with insomnia disorder tend to underestimate TST (as discussed above), this was further investigated in their study by subdividing their population into accurate/overestimators and underestimators, resulting in analysis of relatively small samples. As this is the only study of a adults of a wider age range, further exploration of how sleep perception changes across CBT-I in an adult population with insomnia disorder is warranted.

It has been suggested that those with insomnia disorder may not misperceive sleep only because of poor perception of time, but also because of cognitive and psychological factors.^23^ There is established literature that shows that CBT-I improves psychological symptoms such as depression, anxiety, fatigue, and we were interested in understanding how these changes may or may not correlate with changes in the objective-subjective sleep discrepancy. This may provide additional understanding to how CBT-I works to improve these factors despite the symptoms not being directly targeted by the treatment. In theory, psychological factors such as mood, anxiety, fatigue, or sleep-related cognitions could predispose individuals to altered perception of sleep and serve as a link between how changes in subjective-objective sleep discrepancy may change across treatment. There have been few studies to look at the link between changes in the subjective-objective sleep discrepancy, and those that have were cross-sectional and focused on mood and anxiety.^24,25^ It remains unclear whether psychological variables contribute to the changes in sleep misperception.

Our study aimed to understand how undergoing treatment with CBT-I changes the discrepancy between objective and self-report sleep across a 6-week group CBT-I protocol. The primary objective of this study was to determine how the discrepancy between wrist-worn actigraphy, and sleep diary-derived sleep parameters change across CBT-I. We hypothesized that sleep perception would improve with each subsequent CBT-I session, such that sleep discrepancy decreases across treatment from baseline to post-treatment.

The secondary aim of the study was to understand whether clinical variables including depression, anxiety, fatigue, and maladaptive sleep-related cognitions were associated with a change in the subjective-objective sleep discrepancy across CBT-I treatment. Previous studies have shown that actigraphy is a valid method for measuring sleep parameters in those with comorbid insomnia and depression.^26^ Depressive symptoms have been consistently shown to decrease following CBT-I, but there are mixed results for its impact on fatigue symptoms.^27^ We hypothesized that depressive and anxiety symptoms will decrease and that this will be related to a decrease in the subjective-objective discrepancy as well. As sleep-related are targeted with CBT-I, we predicted that a decrease in dysfunctional beliefs about sleep and fatigue will also be correlated with a decrease in the subjective-objective sleep parameter discrepancies.^21^

## Methods

### Study participants

Twenty-five participants (mean age = 51.93, SD = 14.16, range 22 – 70), with a diagnosis of insomnia disorder were included in the present study. Participants were screened for eligibility for group CBT-I clinical services after being referred from the Sleep Medicine Program, Firestone Institute for Respiratory Health or the Mood Disorders Outpatient Clinic at St. Joseph's Healthcare, Hamilton. Following the screen, participants were invited to participate in research if their symptoms met criteria for insomnia disorder and they were enrolled in group CBT-I. All study participants were informed in detail about the purpose of the investigation and provided their written informed consent prior to the onset of the study. The study was approved by the local ethics committee, the Hamilton Integrated Research Ethics Board.

To determine the sample size of the study, we used the G*power version 3.1.9.6.^28^ A total sample size of 23 was needed for repeated measure analysis, with an alpha error of 0.01, a power of 0.8, an expected medium effect size of 0.25.^29^

### Screening

The presence of insomnia disorder was confirmed based on Diagnostic and Statistical Manual of Mental Disorders (DSM-5) criteria with the Duke Structured Interview for Sleep Disorders (DSISD).^30^ Participants were assessed with the DSISD for the presence of other sleep disorders (e.g., sleep apnea, limb movement, and circadian rhythm disorders) and were excluded if the comorbid sleep disorders were considered primary or were untreated/unstable (e.g., those with sleep apnea who were adherent to positive airway pressure therapy were included). The Mini-International Neuropsychiatric Interview^31^ was conducted to assess participants for comorbid psychiatric disorders. Participants were excluded for presence of bipolar disorder and psychotic disorders (e.g., schizophrenia, schizoaffective disorder). It is important to note that CBT-I has been shown to be effective for those with bipolar or psychotic disorders^32-34^; however, we did not include these individuals in our study due to provision of manualized group CBT-I. Exclusion criteria also included: current shift work, current or recent history (in the past six months) of alcohol or substance abuse or dependence, unmanaged chronic pain that interfered with sleep, and trauma-related nightmares that disrupted sleep. Participants with other comorbid psychiatric and managed medical conditions were otherwise included, since CBT-I for insomnia disorder has been shown to be effective in the context of a range of co-occurring psychiatric conditions.^35^ Prescription and over-the-counter sleep medications were allowed over the duration of the study provided participants remained on a stable dose throughout (i.e., were using the medication non-contingently from screening to post-treatment).

### Study design

The present study employed a within-subject repeated-measures design. Participants completed an initial screening visit to determine eligibility for the study. A baseline assessment was conducted within two weeks prior to initiating group CBT-I along with a battery of measures related to sleep and clinical variables. Actigraphy monitoring was initiated at the baseline visit and continued until the post-treatment visit within two weeks after completing CBT-I. This same battery was administered again at a post-treatment assessment, within two weeks following completion of group CBT-I. Participants were considered completers if they attended four or more of the six group CBT-I sessions. If they missed a session, they were provided with an individual make up session.

### Daily study measures

#### Actigraphy

Objective measures of sleep were obtained using the wrist-worn Philips Respironics Actiwatch Spectrum Plus device. The actigraphy data were collected using 1-minute epochs continuously from the baseline visit, throughout CBT-I, until the post-treatment visit which occurred within two weeks after completing the final treatment session. Actigraphy data was extracted using the Philips Respironics Actiware software (version 6.0). Raw actigraphy reports were visually inspected to identify artefacts and remove missing data (i.e., device removed from wrist) according to published guidelines.^6^ Default sleep/wake thresholds from the software were then used to determine periods of sleep and activity periods. The default wake detection method in the Actiware software was used to detect sleep and rest intervals. The automatic wake threshold method was used, which computes an automatic threshold for wake based on activity counts. The threshold uses the sum of activity counts divided by mobile time multiplied by 0.88.

Visual inspection of the visualization of activity and light data along with the Actiware-detected intervals was used to verify that the sleep intervals were not non-wear, where intervals were removed if there was, for instance, zero activity throughout the duration sleep interval. The Actiware sleep algorithms yields better performance in sleep detection than the Cole-Kripke algorithm, when compared with polysomnography.^36^ The extracted outcomes included the average SOL, WASO, TST, and SE for each period of interest (i.e., baseline, week 1, 2, 3, 4, 5 and post-treatment).

#### Sleep diary

Self-reported measures of sleep were obtained using a sleep diary. Sleep diaries are the gold-standard for tracking sleep disturbances, and the information derived is used to determine sleep efficiency, initiation, and maintenance.^37^ The Consensus Sleep Diary for Morning (CSD-M) is a standardized prospective sleep assessment tool that requires participants to self-report daily estimates of sleep patterns and quality upon waking. Information from the CSD-M was used to calculate sleep parameters, including SOL, WASO, TST, and SE (SE percentage = TST/time in bed). Data were averaged across the week for the CSD-M sleep parameters. Averages were the CSD-M sleep parameters were only calculated if ≥ 5 days of data were recorded for that week. Eligible participants were asked to complete the CSD-M for two weeks prior to treatment, which was used to confirm the presence of insomnia (i.e., SE ≤ 85%). They were asked to continue to use the CSD-M throughout the 6-weekly CBT-I sessions until the post-treatment visit, which occurred within 1-2 weeks after the final treatment session.

### Clinical measures

The Insomnia Severity Index (ISI) is a 7-item self-report scale used to evaluate the severity of insomnia symptoms.^38^ Each item is scored on a 0 to 4 scale with higher values representing greater insomnia severity.^38^ Scores > 11 are indicative of clinical insomnia and an improvement of ≥ 9 was consistent with marked improvement in symptoms.^39^ The ISI has shown high internal consistency in clinical samples (Cronbach's α = 0.91).^39^ In this sample, internal consistency for ISI was good (Cronbach's α = 0.71). This is in line with the literature, which shows that most studies show Cronbach's α ≥ 0.70, but with high heterogeneity across studies.^40^

The Dysfunctional Beliefs About Sleep (DBAS) is a 16-item scale used to assess changes in sleep-related cognitions such as worry, faulty beliefs, and attentional bias across treatment, validated in an insomnia population.^41^ Each item is scored between 0 (strongly disagree) to 10 (strongly agree), and an average score is calculated. Higher scores indicate greater sleep-related dysfunctional cognitions.^41^ The DBAS has strong internal consistency (Cronbach's α= 0.80), concurrent validity, and sensitivity to change following CBT-I.^42^ In this sample, internal consistency was good (Cronbach's α = 0.81).

The Fatigue Severity Scale (FSS) is a 9-item questionnaire that assesses physical tiredness and lack of energy.^43^ Responses are on a 7-point scale ranging from 1 (strongly disagree) to 7 (strongly agree), with higher scores indicating greater fatigue.^43^ It has been shown to measure self-reported fatigue with high internal consistency (Cronbach's α= 0.95).^44^ In this sample, internal consistency for FSS was high (Cronbach's α = 0.94).

The Patient Health Questionnaire-9 (PHQ-9) is a 9-item brief self-report measure of depressive symptoms rated from 0 ("not at all") to 3 ("nearly every day").^45^ A total score of greater than 10 has been shown to have sensitivity of 88% and specificity of 88% for major depression in a primary care setting.^45^ In this sample, internal consistency was high (Cronbach's α = 0.88).

The State-Trait Inventory for Cognitive and Somatic Anxiety (STICSA) is a 21-item self-report measure of anxiety, which includes subscales for both cognitive and somatic anxiety.^46^ Items are rated on a 4-point Likert scale. It has been validated in clinical and non-clinical samples and the subscales have demonstrated excellent internal consistency (Cronbach's α > 0.87).^47^ In this sample, internal consistency was high (Cronbach's α = 0.91).

### Intervention

The CBT-I intervention was a manual-based group intervention that consisted of six two-hour sessions. It was conducted by licensed doctoral clinical psychologists and trained clinical psychology graduate students. The groups consisted of a maximum of six participants. The manual was developed to include components of the intervention such as psychoeducation and sleep hygiene, stimulus control, time in bed restriction, cognitive restructuring, relaxation/counter-arousal strategies, and relapse prevention.^48^ Session-by-session content is included in Table 1. Time-in-bed restriction was conducted based on a minimum time-in-bed of 6 hours and was extended when SE reached 85%. The sleep window was extended by 15 minutes at a time and the positioning of the sleep window was decided collaboratively between the clinicians and participants based on preferences to ensure highest likelihood of adherence.^49^ Stimulus control instructions included the following guidelines^50^: (1) only go to bed when sleepy, but not before earliest prescribed bedtime; (2) not engage in wakeful activities in bed; (3) not take naps during the day; (4) wake up at the same time every day; (5) leave the bed or bedroom if unable to fall asleep at the beginning or middle of the night, and only return when sleepy; (6) if unable to fall back to sleep within 30 minutes, leave the bed or bedroom and only return when sleepy. These stimulus control guidelines were introduced at the first session and reinforced at each subsequent session. Learning was reinforced with weekly home practice exercises, including stimulus control, time in bed restriction, relaxation strategies, and cognitive strategies.

**Table 1:** Description of group CBT-I session content

Session	Content
Session 1	Introduction to CBT, psychoeducation on insomnia, sleep drive, stimulus control, time-in-bed restriction therapy.
Session 2	Review of psychoeducation, time-in-bed restriction therapy, adherence difficulties.
Session 3	Counter-arousal strategies (e.g., relaxation training, ‘worry time’).
Session 4	Review of CBT model, Understanding dysfunctional beliefs about sleep, cognitive restructuring.
Session 5	Continued cognitive restructuring, identifying current obstacles with sleep difficulties, maintaining gains.
Session 6	Relapse prevention, Individualized recommendations.

### Objective and subjective sleep discrepancies

To examine the primary objective of determining how the discrepancy in sleep parameters change across treatment, we first calculated the "discrepancy score" between the actigraphy and sleep diary-derived sleep parameters by subtracting the sleep diary value from the actigraphy value for each sleep parameter (SOL, WASO, TST, and SE). A positive discrepancy score indicated a subjective underestimation of sleep parameters with the sleep diary and a negative score indicated an overestimation with the sleep diary. This is an established measure of sleep discrepancy in the literature.^7,16,19,29^ The discrepancy score was calculated as the difference between the average subjective and objective sleep parameter value for each week. In addition to this absolute discrepancy score, the misperception index (MI) was calculated for TST as a relative measure of discrepancy (objective TST - subjective TST/objective TST), as this is a formula frequently reported for TST misperception.^51^ Positive MI indicates underestimation of objective sleep and negative MI indicates overestimation with the sleep diary.

### Statistical analyses

Statistical analyses were performed using *R* (Version 4.2.2). We first determined whether sleep parameters changed across CBT-I using paired analysis between baseline and post-treatment. The changes in the actigraphy sleep parameters, sleep diary-derived parameters, and discrepancy scores from baseline to post-treatment were determined using either the Wilcoxon or paired t-tests, depending on normality of the data, which we determined using the Shapiro-Wilks test for normality. Effect sizes were determined with Cohen's D.

The primary objective of assessing how the discrepancy in sleep parameters changed across all timepoints (baseline compared to each subsequent sessions and post-treatment) was determined using a linear mixed-effects models (LMM). Four separate models were used with the sleep parameter discrepancy scores (for SOL, WASO, TST, and SE) as the dependent variable and time (i.e., the sessions) as the independent variable. LMMs was used because the data does not meet the criteria of independence due to the within-subject design. LMM allows for regression in which observations are correlated, by including a ‘random effects’ factor to specify that data within each participant is dependent. The Maximum-Likelihood estimation was used. For models with a significant main effect of time, post hoc pairwise comparisons between baseline and each subsequent session, resulting in 6 contrasts (baseline to session 1, 2, 3, 4, 5 and post-treatment). Multiple comparisons were corrected for using the Bonferroni method.

The secondary objective namely, to understand how clinical variables were associated with the discrepancy in sleep parameters was investigated using repeated measures correlations (RMC). This was performed using the rmcorr package in *R* (Bakdash and Marusich, 2017).^52^ This allowed for assessment of how change in each clinical variable was associated with the change of discrepancy between objective and subjective sleep variables from baseline to post-treatment. The RMC takes into account within-participant variation, rather than averaging the repeated measure data from each participant before performing a correlation (which does not meet the assumption of independence). RMC analysis estimates the common regression slope/association between repeated measures that is shared among participants. Non-independence is accounted for by considering each participant as a factor-level variable and therefore removing inter-individual variation. The rho correlation coefficient is bounded by −1 to 1 and represents the strength of the overall or common intra-individual linear relationship between two measures over multiple timepoints.^52^ Multiple comparisons were corrected for using the Bonferroni method (5 clinical variables and 4 sleep discrepancy scores were correlated for a total of 20 tests, therefore the adjusted threshold for significance was set to p < 0.0025).

## Results

Data from 25 participants were included in the final analysis. Two participants completed the final session but stopped recording in their sleep diaries after session 6. For these two participants, the session 5 sleep diary was used to calculate the post-treatment discrepancy (i.e., last observation carried forward [LOCF]). The LOCF approach was used for session 5 since the content of session 6 was relapse prevention and did not include or introduce new content, and therefore the data are comparable for these two participants. Sensitivity analysis revealed that this did not significantly change the results using LOCF. Demographic characteristics are described in Table 2. Changes in sleep parameters and self-report clinical scales across CBT-I are shown in Table 3. Completing CBT-I led to significant reduction in insomnia severity (ISI; t = 10.26, p < 0.01, *d* = 2.31), which reflects a clinically significant improvement (i.e., decrease from moderate insomnia to not clinically significant insomnia). There were also significant improvements in dysfunctional beliefs about sleep (DBAS, t = 8.44, p < 0.01, *d* = 1.75), depressive symptoms (PHQ-9, t = 5.27, p < 0.01, *d* = 0.96), anxiety symptoms (STICSA, t = 3.22, p < 0.01, *d* = 0.46), and fatigue (FSS, t = 6.66, p < 0.01, *d* = 1.08).

**Table 2:** Demographic and clinical characteristics of the sample

Demographics (N=25)		Mean (SD)	Range
Age (years)		52.92 (14.32)	22-70
	Males (n, %)	7 (28%)	
	Females (n, %)	18 (72%)	
Education (total years)		15.44 (2.58)	12-21
Ethnicity (n, %)			
	Arab	2 (8%)	
	Caucasian	21 (84%)	
	Chinese	1 (4%)	
	Black	1 (4%)	
Employment status (n, %)			
	Currently working	11 (44%)	
	Unemployed	2 (8%)	
	Long-term disability	2 (8%)	
	Retired	10 (40%)	
Psychiatric comorbidities (n, %)			
	Major depressive disorder or episode	10 (34%)	
	Generalized anxiety disorder	3 (10%)	
	Social anxiety disorder	4 (14%)	
	Agoraphobia	1 (3%)	
	Post-traumatic stress disorder	1 (3%)	
Duration of insomnia (years)		14 (11)	1.5-45
Comorbid sleep disorders (n, %)			
	Obstructive sleep apnea	9 (31%)	
	Restless legs syndrome	2 (7%)	
	Circadian rhythm disorder	1 (3%)	
	Excessive daytime sleepiness	1 (3%)	
	Periodic limb movement	1 (3%)	
Psychotropic medication use			
	Antidepressants	13 (45%)	
	Antipsychotics	3 (10%)	
	Benzodiazepine hypnotics	7 (24%)	
	Non-benzodiazepine hypnotics	4 (14%)	

**Table 3:** Baseline to post-treatment differences in measures

Measure		Baseline Mean (SD)	Post-treatment Mean (SD)	Paired analysis (t-test [t] or Wilcoxon [V])	Effect size (Cohen's D interpretation)
CSD-M sleep diary					
	SOL (min)	55.27 (50.51)	19.08 (16.93)	V = 324**	0.90 (large)
	WASO (min)	147.31 (84.62)	65.86 (38.85)	V = 313**	1.12 (large)
	SE (%)	70.44 (14.89)	84.24 (9.22)	V = 19.5**	1.10 (large)
	TST (min)	348.26 (88.55)	370.51 (82.25)	t = −1.42	
Actigraphy					
	SOL (min)	18.58 (15.30)	16.30 (15.74)	V = 163	
	WASO (min)	54.56 (19.64)	45.94 (17.16)	t = 2.21*	0.44 (small)
	SE (%)	83.09 (5.80)	82.85 (7.86)	V = 176.5	
	TST (min)	413.90 (76.15)	379.34 (88.72)	t = 4.19**	0.84 (large)
Self-report measures					
	ISI	18.82 (4.26)	7.56 (5.15)	t = 10.26**	2.31 (large)
	DBAS	5.98 (1.38)	3.04 (1.93)	t = 8.44**	1.75 (large)
	FSS	4.56 (1.50)	3.01 (1.35)	t = 6.66**	1.08 (large)
	PHQ-9	9.56 (5.53)	4.84 (4.19)	t = 5.27**	0.96 (large)
	STICSA	39.72 (10.70)	35.28 (8.54)	t = 3.22**	0.46 (small)

DBAS = Dysfunctional Beliefs About Sleep; FSS = Fatigue Severity Scale; ISI = Insomnia Severity Scale; PHQ-9 = Patient Health Questionnaire-9; SE = sleep efficiency; SOL = sleep onset latency; STICSA = State-Trait Inventory for Cognitive and Somatic Anxiety; TST = total sleep time; WASO = wake after sleep onset.

N = 25;

* p < 0.05,

** p < 0.001; Cohen's d interpretation: 0.2 = small effect size, 0.5 = medium effect size, 0.8 = large effect size.

Paired-analyses from baseline to post-treatment of the sleep diary and actigraphy-derived sleep parameters are shown in Table 3. Self-reported sleep variables, derived from sleep diaries, all showed significant improvement across CBT-I. Self-reported SOL was significantly shorter (V = 324, p < 0.01, *d* = 0.90), self-reported WASO significantly decreased (V = 313, p < 0.01, *d* = 1.12), and the SE was significantly higher (V = 19.5, p < 0.01, *d* = 1.10), all with large effect sizes. The self-reported TST did not significantly change (t = −1.42, p = 0.17). Actigraphy-derived objective sleep parameters showed a reduction in WASO with a small effect size (t = 2.21, p = 0.04, *d* = 0.44), and TST decreased with a large effect size (V = 4.19, p < 0.01, *r* = 0.84). There were no significant changes in objective SOL (V = 163, p = 0.72). and SE (V = 176.5, p = 0.72) between baseline and post-treatment.

**Figure 1:**
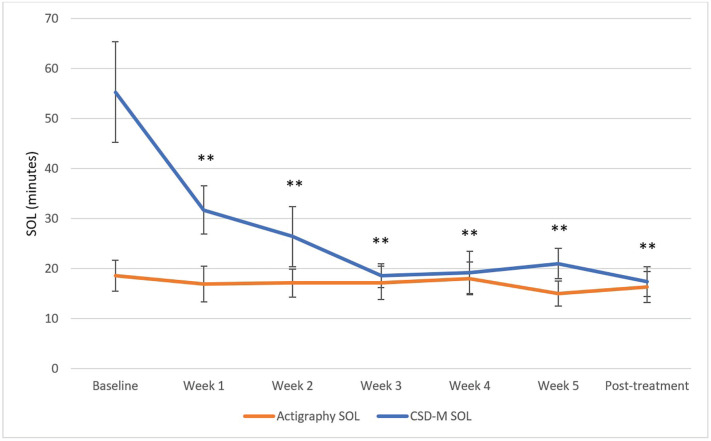
Mean SOL measured with actigraphy and sleep diary across CBT-I sessions. CSD-M = Consensus Sleep Diary - Morning; SOL = sleep onset latency. * p ≤ 0.05, ** p ≤ 0.0025 (Bonferroni correction) for discrepancy comparison with baseline.

**Figure 2:**
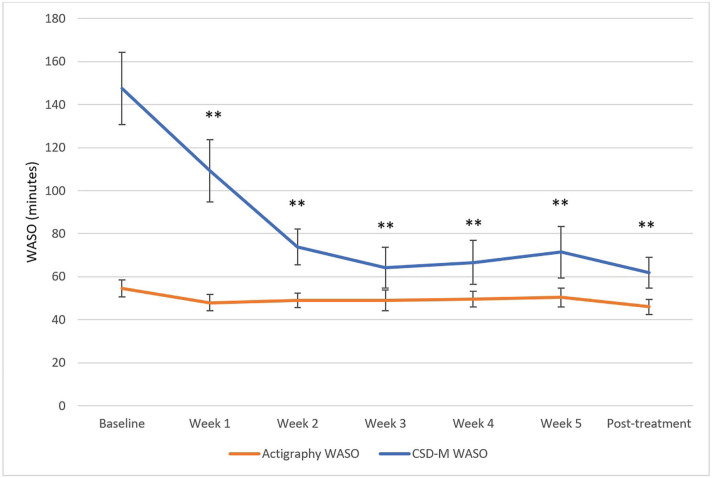
WASO measured with actigraphy and sleep diary across CBT-I sessions. CSD-M = Consensus Sleep Diary - Morning; WASO = wake after sleep onset. * p ≤ 0.05, ** p ≤ 0.0025 (Bonferroni correction) for discrepancy comparison with baseline.

**Figure 3:**
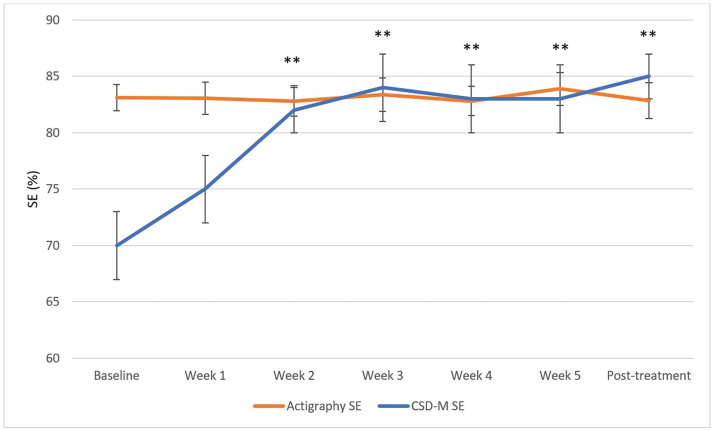
SE measured with actigraphy and sleep diary across CBT-I sessions. CSD-M = Consensus Sleep Diary - Morning; SE = sleep efficiency. * p ≤ 0.05, ** p ≤ 0.0025 (Bonferroni correction) for discrepancy comparison with baseline.

**Figure 4:**
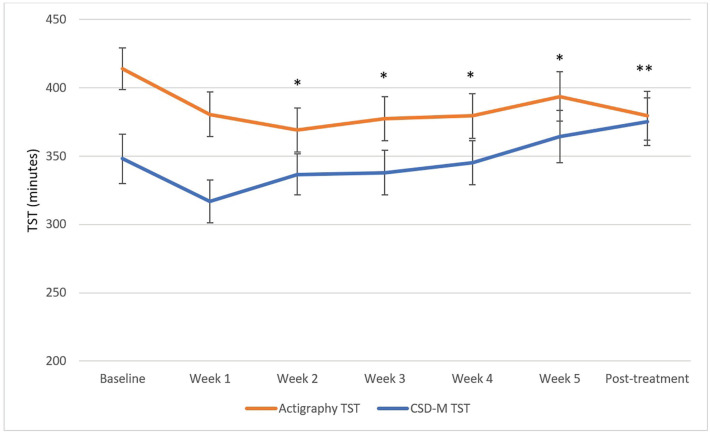
TST measured with actigraphy and sleep diary across CBT-I sessions. CSD-M = Consensus Sleep Diary - Morning; TST = total sleep time. * p ≤ 0.05, ** p ≤ 0.0025 (Bonferroni correction) for discrepancy comparison with baseline.

**Figure 5:**
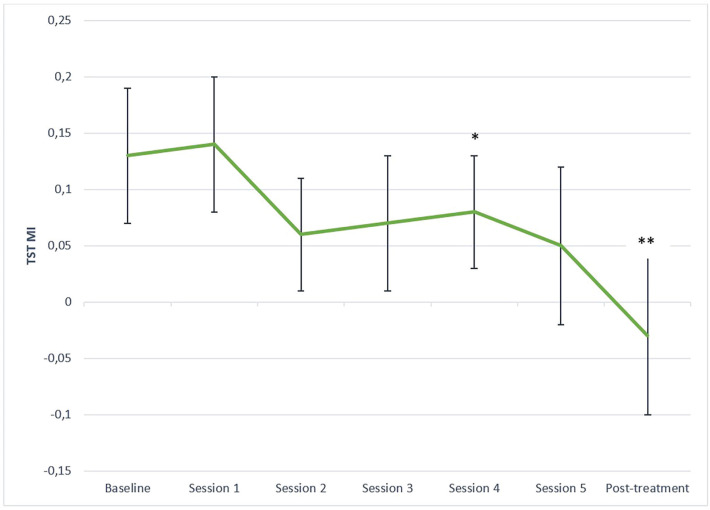
Misperception index (MI) for total sleep time across CBT-I sessions. CSD-M = Consensus Sleep Diary - Morning; MI = misperception index; TST = total sleep time. * p ≤ 0.05, ** p ≤ 0.0025 (Bonferroni correction) for MI comparison with baseline.

The primary aim was to evaluate how the discrepancy scores between self-reported and actigraphy-derived sleep parameters changed across CBT-I using LMMs. The LMMs revealed significant reduction in the discrepancy scores between baseline and each of the 6 subsequent sessions for the SOL discrepancy scores (χ²(6) = 45.12, p < 0.001) and the WASO discrepancy scores (χ²(6) = 75.94, p < 0.0001). The SE discrepancy scores (χ²(6) = 58.71, p < 0.001) significantly decreased between baseline and session 2, 3, 4, 5, and post-treatment (i.e., all contrasts except between baseline and session 1). The absolute TST discrepancy scores (χ²(6) = 28.86, p < 0.001) significantly decreased from baseline to post-treatment following correction for multiple comparisons (Bonferroni method). The LMM revealed that the relative TST MI scores significantly decreased between baseline and post-treatment only (χ²(6) = 27.23, p < 0.001) following Bonferroni correction. The mean MI was positive at baseline and negative at post-treatment. These results are illustrated in Figures 1-5. Please note, for Figures 1-4, showing means and standard error bars, that the discrepancy is reflected by the distance between the lines which plot the sleep diary and actigraphy-derived sleep parameters in each graph, such that larger gaps between the lines indicate a greater discrepancy.

The secondary analysis of how change in discrepancy scores was related to change in clinical variables are reported in Table 4. The direction of the correlation was related to *how* the discrepancy changed across CBT-I. For example, sleep diary SE and TST values were both lower than the actigraphy values at baseline and increased following treatment; whereas the sleep diary SOL and WASO values were higher than the actigraphy values at baseline and decreased following treatment. The direction of this change is the direction of the RMC results (positive for SE/TST and negative for SOL/WASO). The RMC analyses revealed that there were significant associations between the change in WASO discrepancy and several self-report clinical variables including insomnia severity (ISI *r* = −0.60), depression (PHQ-9 *r* = −0.61), and dysfunctional beliefs about sleep (DBAS *r* = −0.61) after correction for multiple comparisons. It was not significantly correlated with fatigue (FSS *r* = −0.52) after correction for multiple comparisons, or anxiety (STICSA *r* = −0.45). The change in SE discrepancy scores were also significantly correlated with insomnia severity (ISI *r* = 0.72), depression (PHQ *r* = 0.60), fatigue (FSS *r* = 0.57), and dysfunctional beliefs about sleep (DBAS *r* = 0.68) after correction for multiple comparisons, but not anxiety (STICSA *r* = 0.46). The RMC results showed that the change in discrepancy for SOL and TST were not correlated with the specified clinical variables measured.

**Table 4:** Repeated measures correlations between baseline and post-treatment sleep discrepancy scores and clinical self-report variables

Correlations (*r*)	SOL discrepancy	WASO discrepancy	SE discrepancy	TST discrepancy
ISI	-0.56*	-0.60**	0.72**	0.47
PHQ-9	-0.44	-0.61**	0.60**	0.45
STICSA	-0.23	-0.45	0.46	0.37
FSS	-0.40	-0.52*	0.57**	0.50*
DBAS	-0.41	-0.61**	0.68**	0.52

* p < 0.05,

** Bonferroni corrected p < 0.0025.

## Discussion

Our study aimed to investigate the change in subjective-objective sleep discrepancy across CBT-I using a sleep diary and wrist-worn actigraphy device. Our analyses revealed a significant decrease in the discrepancy between subjective and objective measures of sleep following CBT-I. This change occurred after a single session for SOL and WASO, and after the second session for TST and SE, and persisted throughout the course of the CBT-I treatment. This outcome could be explained as an improvement in sleep perception that was observed early on in treatment and was maintained by post-treatment. At baseline, our study showed a significant mismatch between sleep-diary and actigraphy. This was expected based on previous cross-sectional studies,^53^ which showed that those with insomnia disorder tended to overestimate time to fall asleep, length of awakenings, and underestimate the duration of sleep. Previous studies that investigated the subjective-objective sleep discrepancy with actigraphy as the objective measure also found the discrepancy significantly decreases following CBT-I.^15,18,19,22^ However, many of the studies of the change in subjective-objective sleep discrepancy across CBT-I have focussed on older adults (i.e., over age 55) only.^16,18-21^ Our results extend these findings by showing the decrease in discrepancy occurs in a group of treatment-seeking adults with insomnia disorder with a wide age range (ages 22-70 included), and not only in older adults. This is an important replication of the single study which has also found decreased discrepancy in adults across CBT-I.^22^

The changes in the discrepancy scores were significant for each of the sleep parameters in our study (SOL, TST, WASO, SE) and could be driven by several factors, including more awareness of sleep as participants gain more experience with documenting multiple aspects of their sleep experience with the sleep diary over time. Importantly, the initial session of CBT-I introduces critical psychoeducation on insomnia, including the diagnostic criteria, stimulus control, and rationale for time-in-bed restriction. During the first session participants are asked to pay attention to subtle changes within their 24-hour sleep experience and document this on the sleep diary throughout the course of CBT-I. As a result, the decrease in sleep discrepancy may be seen as a ‘correction’ in sleep perception. However, this alone is unlikely to account for the significant change in discrepancies between baseline and the first session, since participants had already filled out the sleep diary for 1-2 weeks prior to initiating treatment. The sleep diary is a necessary tool for effectively implementing time in bed restriction and stimulus control,^3^ since clinicians base personalized recommendations for time into and out of bed based on the behaviours from the previous week as recorded on the sleep diary. Therefore, sleep diary reported sleep parameters reflect both the behavioural changes and perceptual changes across treatment. In theory, actigraphy should also capture behaviours that encompass stimulus control and time in bed restriction (i.e., movements of getting into/out of bed upon awakenings, with an emphasis on getting out of bed if awake for more than 20 minutes), but not perception. Since psychoeducation occurs alongside behavioural changes of stimulus control and time-in-bed restriction, we are unable to disentangle which components are driving this early change in subjective-objective sleep discrepancy.

Our results showed that the TST discrepancy significantly decreased from baseline to post-treatment. Two recent studies also showed that the discrepancy in TST significantly improved with CBT-I.^15,54^ This trend began in our study following the first session, when time in bed restriction and stimulus control strategies were delivered, with adjustments to implementing these strategies made in subsequent sessions based on sleep diary analysis and problem solving. A recent meta-analysis analyzed how sleep duration changed across CBT-I measured with PSG, actigraphy, and sleep diary.^55^ TST significantly increased by an average of 30 minutes following CBT-I when measured with sleep diary and PSG. Interestingly, actigraphy-measured TST significantly decreased by an average of 30 minutes following CBT-I. Indeed, this is the trend within our study, as TST measured by the sleep diary increased while TST measured by actigraphy decreased from baseline to post-treatment. The relative change in TST, as measured with the MI, showed a trend from underestimation of TST with sleep diary at baseline to being accurate or overestimating TST across sessions, and was significantly decreased by post-treatment. This significant decrease in MI was similar to the results shown by Janku et al.,^22^ except their total sample mean MI was accurate at baseline and were overestimators of TST following CBT-I. This suggests our sample had a greater proportion of participants who underestimated their sleep and that this sleep misperception was corrected across CBT-I.

Another possible explanation for why the subjective-objective sleep discrepancy decreased following CBT-I, is that actigraphy is better able to capture sleep as participants change their behaviours during treatment. Actigraphy has been shown to overestimate total sleep time compared to other objective measures of sleep, such as PSG.^56^ A drawback of using actigraphy as an objective marker of sleep is that it only provides an indirect measure of sleep based on lack of movement, so a participant who is still in bed may be inaccurately scored as asleep even if they are awake.^57^ This suggests that rather than there being a "misperception" of sleep, participants may be accurately reporting sleep, while actigraphy is overestimating sleep parameters. This may be especially true at baseline, when participants are more likely to be lying in bed awake, prior to the introduction of stimulus control. A recent study found that the mean amount of misperception of sleep between actigraphy and sleep diary was able to differentiate those with insomnia disorder from healthy controls, which suggests that the difference in these measures is capturable prior to treatment.^58^ In summary, it is possible that actigraphy is better able to capture the "awake time" during and after CBT-I, which may be a driving force of the decreased sleep discrepancy.

The secondary analyses in this study investigated how the changes in subjective-objective sleep discrepancy were related to changes in clinical variables across treatment. The decrease in depression symptoms, dysfunctional beliefs about sleep, fatigue symptoms and insomnia symptom severity overall were significantly correlated with changes in WASO, SE, TST and SOL. These results suggest that there is a correlation in the degree of clinical symptom change alongside the improvement in sleep discrepancy. Depression, fatigue, and negative beliefs about sleep may bias the sleep diary reports at baseline and improvement in these clinical symptoms may help with the ‘correction’ of the discrepancy by post-treatment. Interestingly, the change in anxiety (as measured with the STICSA) was not correlated with any of the changes in sleep discrepancies, possibly because the degree of change was smaller in this measure compared to the other clinical variables. As these are correlational results, one drawback is that causation of these changes cannot be implied. A future direction of this study is to look at subsets of a larger sample to understand the direction of change (i.e., how sleep discrepancy changes in those with greater depression compared to lower depression scores).

Actigraphy-measured sleep parameters changed less than sleep diary-measured parameters in our study, which is in line with the established literature.^22^ A recent study found that based on at-home actigraphy, most sleep variables did not differentiate insomnia from controls.^59^ This study also suggested that the within-subject night-to-night variability in SE and WASO differentiated those with insomnia disorder from healthy controls, but only with small effect sizes.^59^ A possible future direction of our study is to evaluate whether CBT-I leads to a decrease in the night-to-night variability in sleep parameters rather than the average of each sleep parameter over each week.

There are important implications for how these results could be used therapeutically. For example, Tang and Harvey^60^ showed that patients who were shown the discrepancy between self-reported sleep estimates and actigraphy recordings had improved anxiety and more accurate SOL estimates. Similarly, use of actigraphy could prove important for showing participant's perceptual differences across treatment. Incorporating feedback from sleep wearables into CBT-I treatment, including the subjective-objective sleep discrepancy, is an important future direction of this work. A recently published study protocol aims to use wrist-worn devices to provide feedback to the participant throughout a randomized controlled trial for CBT-I.^61^ The use of devices has become increasingly popular but current treatments do not routinely use data from objective sleep during treatment. As we have shown in this study, the objective measures of sleep do not match subjective experiences prior to the start of treatment; however, exploring this discrepancy with participants at the start and during treatment has the potential to further improve outcomes within the first few weeks of intervention.

Understanding the neurobiology of insomnia, and in particular, the tendency to underestimate sleep duration/misperceive sleep, is another important future direction of this work. The brain activity of those with insomnia showed there was higher activation with EEG in NREM sleep compared to healthy controls.^62^ In addition, the density of sleep spindles appears to differ in those with "paradoxical" insomnia, suggesting a possible neurobiological basis for an increased subjective-objective sleep discrepancy.^63^ It is possible that those with insomnia require a greater amount of sleep in order to perceive the state of sleep, which may explain why the objective and subjective experiences of sleep are different for the insomnia population.^64^ This has been shown for misperception of SOL, since those with insomnia required 34 minutes of undisturbed sleep before recognizing sleep onset, compared to 22 minutes for healthy controls.^64^ These studies suggest that those with insomnia may be perceiving more "wake-like" brain activity rather than underestimating their sleep,^62^ and perhaps this is what changes after receiving CBT-I treatment.

There are several limitations of the current study including the lack of a control group to compare our sample to (e.g., those with insomnia undergoing a different treatment, or a wait-list control group). Therefore, we cannot be certain that the changes observed are due to undergoing CBT-I compared to general changes over time. This has been the case with other similar studies,^22^ which suggests that another future direction of treatment should include observing sleep discrepancies across different treatments for insomnia. In addition, our study had a relatively small sample size which limited our ability to look at subsets of our population (e.g., age groups) to further explore factors related to sleep discrepancy. We enrolled participants with other sleep disorders but only if the other sleep disorders were appropriately managed; specifically, if obstructive sleep apnea (OSA) was treated with regular use of continuous positive airway pressure. There is a possibility that those with comorbid insomnia and OSA may respond differently than those with only insomnia.^65^ Our sample captures a "real world" sample from a treatment-seeking population in a tertiary care setting as we aimed to have increased external validity and is in line with the finding that 30-50% of patients in a sleep clinic have comorbid insomnia and OSA.^65^ We also included those who were taking sleep medications, and so future studies are needed to investigate how sleep medication use impacts sleep discrepancies across treatment.

To conclude, the present study was the first to assess the impact of CBT-I on the objective and subjective sleep discrepancies outside of older adults and evaluate the associations with change in clinical variables across CBT-I. The subjective-objective sleep discrepancy significantly improved for all sleep parameters in those with insomnia following CBT-I. This change in discrepancy was ‘corrected’ early on in treatment and was maintained throughout the course of treatment. This suggests that psychoeducation and the early implementation of behavioural components like stimulus control and time in bed restriction, are helpful in changing the mismatch between actigraphy and self-reported sleep. The changes in sleep discrepancy parameters across CBT-I were also associated with the improvement of several clinical variables, such as depression symptoms, fatigue symptoms, dysfunctional beliefs about sleep, and insomnia symptoms in general. Important future directions of this research include using actigraphy as an objective sleep measure to inform participants across treatment, and further exploring the treatment components and clinical factors that influence the sleep discrepancies across CBT-I treatment.
